# Anxiety, Depression, and Stress Are Associated With Internet Gaming Disorder During COVID-19: Fear of Missing Out as a Mediator

**DOI:** 10.3389/fpsyt.2022.827519

**Published:** 2022-02-11

**Authors:** Yang Wang, Bingjie Liu, Lei Zhang, Peng Zhang

**Affiliations:** ^1^Faculty of Psychology, Tianjin Normal University, Tianjin, China; ^2^The School of Psychology and Cognitive Science, East China Normal University, Shanghai, China; ^3^Department of Psychology, Qingdao Academy, Qingdao, China

**Keywords:** depression, anxiety, stress, fear of missing out, internet gaming disorder, teenagers

## Abstract

**Background:**

Many teenagers suffered negative emotional states, especially anxiety and depression, during the COVID-19 outbreak, and most teenagers choose Internet games to cope with negative emotion. Previous evidence indicated that fear of missing out is related with anxiety and depression in teenagers with Internet gaming disorder, but it is unclear how fear of missing out influences depression, anxiety, or stress.

**Methods:**

Based on an I-PACE model, using Depression, Anxiety, Stress Scale (DASS-21), Fear of Missing Out Scale, and Internet Gaming Addiction Scale, and 324 middle school students as participants, this study explored the mediating effect of fear of missing out on depression, anxiety, or stress and Internet gaming disorder.

**Results:**

The results showed that depression and stress are significantly related to Internet gaming disorder through the partial mediating of fear of missing out. Anxiety is not significantly related to Internet gaming disorder through the full mediating of fear of missing out, and anxiety and stress have a greater predictive effect on Internet gaming disorder through fear of missing out. Results also demonstrated that students who play Honor of Kings or Player Unknown's Battlegrounds have more risk to develop Internet gaming disorder.

**Conclusions:**

The results indicated that fear of missing out as a mediator regulates the relationship among depression, anxiety, and stress and Internet game disorder. Specifically, under the mediation of fear of missing out, teenagers with anxiety are more likely to develop Internet gaming disorder, while teenagers with depression or stress might be prone to other types of Internet use disorders.

## Introduction

The outbreak of COVID-19 has had negative influences on the living state and mental health of teenagers. For the prevention and control of the epidemic, social interaction was forced to transfer online from offline (1), and it induced some negative emotions such as anxiety, depression, and stress (2, 3). To relieve negative emotion, most teenagers choose Internet games to cope. In May 2020, *Research Report on the Internet Usage of Minors in China in 2019* released by CNNIC showed that 61.0% of netizens under 18 years old often play Internet games. Listening to music and playing games are the most important entertainment activities for adolescents (4). Adolescents are in a “storm period” of emotional development due to their immature mind and lack of ability for emotional regulation (5). At the same time, adolescents find it easier to relieve negative emotion and obtain satisfaction via the Internet when they are facing troubles in their daily life, and with the undeveloped self-control ability, they are a highly susceptible group for Internet gaming disorder (6).

Internet gaming disorder is classified both in the DSM-5 and ICD-11, defined by an individual who partakes in continuous or repeated gaming (whether online or offline), manifested through impaired control of the game, increased emphasis on the game, and continuous or upgraded gaming regardless of negative consequences (7). Previous evidence showed that anxiety and depression are related to Internet gaming disorder (8–12). It is possible that individuals with depression or anxiety are more likely to socialize on online platforms to meet their psychological needs (13–15). So, the individuals with severe depression and individuals with general anxiety are more likely to overuse smartphones or Internet games (16). Based on previous evidence, the current study proposed that anxiety, depression, and stress are related to Internet gaming disorder (Hypothesis 1). Meanwhile, previous studies also found that demographic variables like age and gender are related to Internet gaming disorder or other problematic use disorders (17, 18). This study explored the relationship among depression, anxiety, stress, and Internet gaming disorder by controlling demographic variables.

Meta-analysis indicated that depression or anxiety is not the only factor influencing Internet gaming disorder; there are other factors between anxiety, depression, stress, and Internet gaming disorder (17, 19). One study focused on adults with Internet gaming disorder and found that individuals with Internet gaming disorder had lower resilience, higher perceived stress, and higher levels of depression. A survey of 812 participants found that the Fear of Missing Out mediated social anxiety and Internet gaming disorder (19). During the period of quarantine, most people got information through the Internet (1), while the flood of information online caused anxiety and caused people to develop a fear of missing out on the information (20).

Fear of Missing Out (FoMO) refers to a pervasive apprehension that others might be having rewarding experiences from which one is absent, characterized by the desire to stay continually connected with what others are doing (20). Studies about FoMO suggested that FoMO involves an adverse negative emotional state, and individuals with high anxiety or depression are more likely to experience FoMO (21, 22). Other studies found that FoMO is associated with higher levels of Internet gaming disorder severity (23, 24), and FoMO mediated relations between depression and Internet gaming disorder severity (16). As we can see in the study by Elhai's team (18), FoMO significantly mediated relations between anxiety and both smartphone use frequency and problematic smartphone use severity, but did not account for relations between depression and smartphone use or problematic smartphone use (18). And although previous studies focused on the mediating effect of FoMO, most of them explored the links between FoMO and Internet gaming disorder in Western culture (19, 25). It is therefore unclear whether FoMO has the same mediating effect in a Chinese context. Additionally, most studies on FoMO explored the level of participants' FoMO through the scale established by Przybylski et al., however, the FoMO Scale from Przybylski et al. was developed from the Western context, and needs to verified in the context of China.

Brand's team (26, 27) proposed an interaction of Person-Affect-Cognition-Execution (I-PACE) model to explain the development of addictive behaviors. I-PACE model believes that different core characteristics have different cognitive biases to the environment, which will affect the first choice of behavior and develop into specific addictive behaviors (26, 27). Though previous research showed that there are mediating factors (like FoMO) between anxiety, depression, stress, and Internet gaming disorder (17, 19), few studies distinguished the relationship among the relationships in anxiety, depression, and Internet gaming disorder, and few studies explored the influence mechanism of the first choice of behaviors. Therefore, this study aims to explore whether the difference exists in the relationship between depression, anxiety, or stress and Internet gaming disorder, and to explore how fear of missing out influences depression, anxiety, or stress.

**Figure 1:**
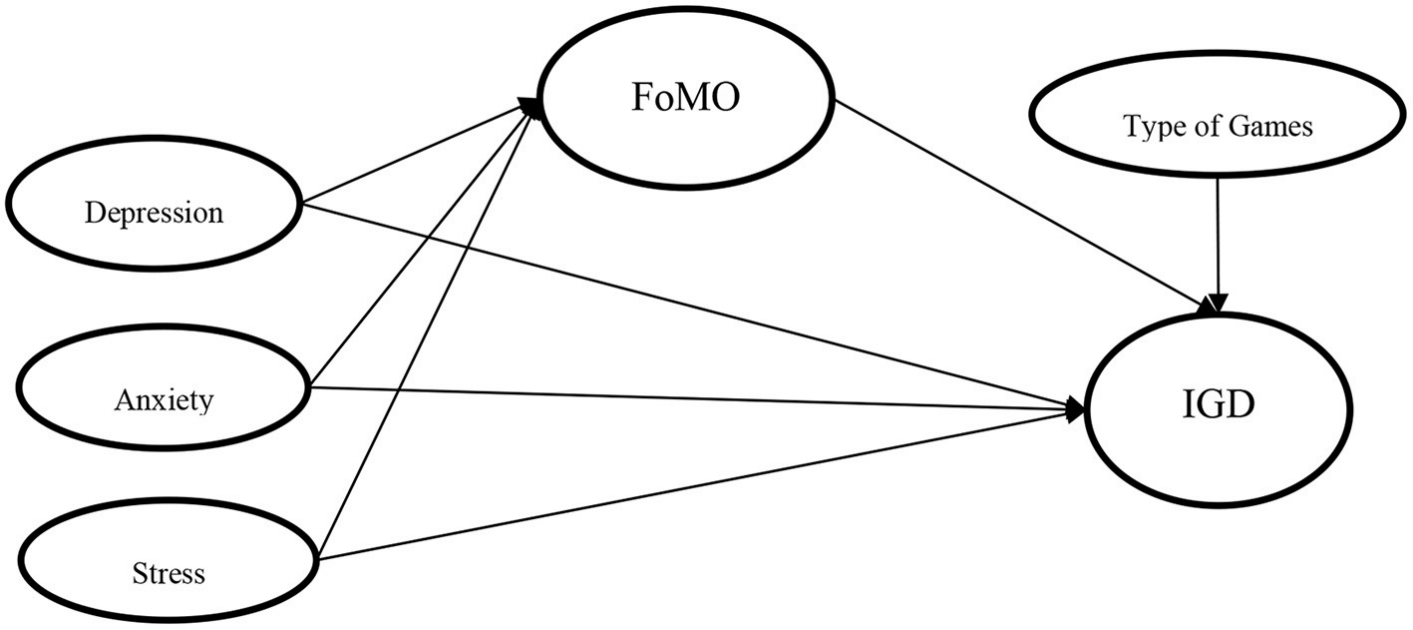
Relation diagram of various variables. FoMO, fear of missing out; IGD, Internet Gaming Disorder.

To explore how FoMO influences the relationship between depression, anxiety, or stress and Internet gaming disorder severity (the mechanism of the first choice of behaviors) and to verify whether the FoMO scale is valid in the context of China, this study used the I-PACE model to explore the mechanism between them. The I-PACE model indicated that addictive behaviors are the consequence of interactions between the core characteristics of a person and several moderating and mediating variables, which may be dynamic and develop over time as a consequence of engagement in specific behaviors (26, 27). So FoMO, as a cognitive factor, is a mediating factor in personal variables and problematic use (26, 27). Specifically, the psychopathological factors (like anxiety, depression, and stress) are mediated by FoMO to affect behavioral decision-making, forming the different addiction behaviors (26, 27). The current study proposed that FoMO is significantly related to anxiety, depression, stress, and Internet gaming disorder (Hypothesis 2), and FoMO mediates the relationship among anxiety, depression, stress, and Internet gaming disorder (Hypothesis 3). The relationship of all variables is shown in Figure 1.

## Methods

### Participants and Procedure

Due to the restriction of mobile phone use in teenagers, participants were randomly recruited from a middle school in Tianjin through convenience sampling in 2021. Specifically, the psychology teacher issued the questionnaires in class and 400 adolescents completed the questionnaire; 324 of them are valid questionnaires. Among the 324 participants, there were 163 boys (50.3%) and 161 girls (49.7%), age range from 12 to 15 years (M ± SD = 13.07 ± 0.76). To explore the distribution of the data, we used the Q-Q graphical method in the SPSS tool to analyze and found that the participants distribution was normal, and subsequent data analysis could be carried out. All the participants volunteered for the study and all the participants and their parents signed their informed consent. Emotional or psychological support was provided when participants wanted it. Ethics approval was given from the Ethics Committee of Tianjin Normal University. The procedures used in this study adhere to the tenets of the Declaration of Helsinki.

### Measures

#### The Depression Anxiety Stress Scale-21 (DASS-21)

In this study, the Chinese version of DASS-21 (28) was used to evaluate the level of anxiety, depression, and stress of the participants. It is a 3-point self-evaluation scale and requires participants to score each item based on their own feelings in the past week (0 = completely inconsistent, 3 = completely in line). The scale consists of three subscales: depression, anxiety, and stress. The higher the score, the higher the level of anxiety, depression, and stress. Previous studies have indicated that DASS-21 has good reliability and validity. In the current study, the Cronbach's α of the depression subscales, anxiety subscales, and stress subscales were 0.87, 0.84, and 0.86.

#### Internet Gaming Disorder Scale

This study uses the Internet gaming disorder scale developed by Cui (29). Cui developed the Internet gaming disorder scale based on DSM-4 and the eight criteria for Internet disorder from Young (29), which consists of 10 items, each with two options of “yes” and “no.” Answering “yes” gets 1 point, while answering “no” gets 0 points. Those with a total score of 7 or >7 are considered as having Internet gaming disorder. The scale is a standard-referenced test prepared by the Angoff method and has good empirical validity (29). We revised the scale based on DSM-5. In order to validate the scale, we issued 300 questionnaires to a middle school in Tianjin through convenient sampling in 2021 and collected 253 valid questionnaires before the formal study. The results of confirmatory factor analysis using AMOS showed that χ^2^/df = 2.85, NFI = 0.88, CFI = 0.90, TLI = 0.87, RMSEA = 0.08. In the current formal study, the Cronbach's α of the Internet gaming disorder scale was 0.75.

#### Fear of Missing Out Scale

In this study, the single-dimensional FoMO scale developed by Przybylski et al. (20) was used to assess the FoMO level of participants. It has 10 items and Likert 5-point scoring (1 = completely non-conforming, 5 = completely conforming) scale. The higher the score, the higher the FoMO level. The initial internal consistency coefficient of this scale is α = 0.87 (19). In order to validate the scale in the context of China, we revised the scale into Chinese by translating it from English to Chinese and back to English again. And then we issued 250 questionnaires to a middle school in Tianjin through convenience sampling in 2020 and collected 203 valid questionnaires before the formal study. The results of confirmatory factor analysis using AMOS showed that χ^2^/df = 2.41, NFI = 0.91, CFI = 0.94, IFI = 0.86, RMSEA = 0.08. It means that the scale has a good validity in the context of China. In this formal study, the Cronbach's α of the scale was 0.84.

### Statistical Analyses

The data were analyzed through SPSS 22.0 for descriptive and correlation analysis, using One-way ANOVA to test the demographic differences of each variable, and used model 4 in the PROCESS 3.3 plug-in to analyze the mediating effect of the model. The scores of each scale are standardized and then further calculated.

## Results

### The General Characteristics of Adolescents' Internet Gaming Disorder

Descriptive statistics and difference tests showed (Table 1) that there are significant gender differences in Internet gaming disorder, but no significant gender differences in depression, anxiety, stress, and FoMO, The scores of Internet gaming disorder in males are significantly higher than in females. There is no significant age difference in depression, anxiety, stress, FoMO, and Internet gaming disorder. There are significant grade differences in depression, anxiety, and stress, but no differences in FoMO and Internet gaming disorder. The score of depression, anxiety, and stress in 8th grade students are significantly higher than in 7th grade students. In terms of the duration of playing a game, there are significant differences in depression, anxiety, stress, FoMO, and Internet gaming disorder. The scores of depression, anxiety, stress, FoMO, and Internet gaming disorder of adolescents with long game duration are significantly higher than adolescents with short game duration. There are no significant differences of type of games in depression, anxiety, stress, and FoMO, but there is significant difference in Internet gaming disorder. The scores of Internet gaming disorder in students who played Honor of Kings or Player Unknown's Battlegrounds are significantly higher than students who played single-player games or do not play.

**Table 1:** Descriptive statistics (M ± SD) and Difference tests of each variable.

**Demographic variable**		**Stress**	**Anxiety**	**Depression**	**FoMO**	**IGD**
Gender	Male	0.62 ± 0.67	0.57 ± 0.64	0.37 ± 0.55	2.11 ± 0.79	0.22 ± 0.21
	Female	0.76 ± 0.72	0.66 ± 0.67	0.49 ± 0.64	2.25 ± 0.86	0.14 ± 0.18
	**t**	**1.84**	**1.27**	**1.81**	**1.43**	−3.58***
Age	12	0.73 ± 0.66	0.64 ± 0.65	0.38 ± 0.49	2.31 ± 0.89	0.17 ± 0.22
	13	0.66 ± 0.69	0.57 ± 0.62	0.42 ± 0.58	2.17 ± 0.78	0.18 ± 0.20
	14	0.69 ± 0.76	0.65 ± 0.72	0.49 ± 0.67	2.03 ± 0.84	0.19 ± 0.19
	15	0.81 ± 0.71	0.94 ± 0.83	0.61 ± 0.85	2.51 ± 0.82	0.26 ± 0.22
	**F**	**0.24**	**1.22**	**0.70**	**1.97**	**0.71**
Grade	7 Grade	0.62 ± 0.60	0.53 ± 0.56	0.35 ± 0.48	2.13 ± 0.78	0.17 ± 0.20
	8 Grade	0.78 ± 0.81	0.74 ± 0.76	0.55 ± 0.71	2.25 ± 0.88	0.19 ± 0.20
	**t**	−2.05*	−2.88***	−3.04***	**−1.25**	**−0.61**
Game duration (week)	<5 h	0.62 ± 0.68	0.56 ± 0.61	0.37 ± 0.51	2.12 ± 0.79	0.14 ± 0.18
	5–10 h	0.78 ± 0.64	0.71 ± 0.64	0.47 ± 0.61	2.21 ± 0.81	0.30 ± 0.19
	>10 h	1.22 ± 0.70	1.04 ± 0.90	0.95 ± 0.87	2.74 ± 1.13	0.41 ± 0.24
	**F**	7.08***	5.12**	8.63***	4.65**	32.11***
Type of games	Honor of Kings	0.67 ± 0.65	0.62 ± 0.64	0.44 ± 0.61	2.14 ± 0.87	0.22 ± 0.22
	PUBG	0.74 ± 0.75	0.57 ± 0.65	0.43 ± 0.55	2.09 ± 0.84	0.21 ± 0.22
	Single games	0.71 ± 0.71	0.65 ± 0.69	0.45 ± 0.61	2.22 ± 0.77	0.16 ± 0.18
	Don't play	0.45 ± 0.60	0.51 ± 0.57	0.34 ± 0.62	2.31 ± 0.88	0.06 ± 0.13
	**F**	**1.07**	**0.45**	**0.22**	**0.66**	5.83***

*p < 0.05, **p < 0.01, ***p < 0.001; FoMO, fear of missing out; IGD, Internet Gaming Disorder; PUBG, Player Unknown's Battlegrounds. Bold values indicate the results of difference tests.

### Correlation Analysis Results

The Correlation Analysis is shown in Table 2. The results showed that there is a positive correlation among depression, anxiety, stress, FoMO, and Internet gaming disorder after adjusting for gender, age, grade, game duration, and type of games. Like the data shows in Table 2, FoMO was moderately correlated with stress, anxiety, and depression scores. So, we estimated Pearson correlations and found that the correlation coefficient of FoMO and stress is 0.58 (*p* < 0.001), the correlation coefficient of FoMO and anxiety is 0.56 (*p* < 0.001), and the correlation coefficient of FoMO and depression is 0.51 (*p* < 0.001). FoMO was just mildly correlated with IGD, with the correlation coefficient of FoMO and IGD being 0.27 (*p* < 0.001).

**Table 2:** Correlation Analysis of each variable.

		**1**	**2**	**3**	**4**	**5**
1	FoMO	1				
2	IGD	0.27***	1			
3	Stress	0.58***	0.26***	1		
4	Anxiety	0.56***	0.26***	0.80***	1	
5	Depression	0.51***	0.26***	0.77***	0.76***	1

*** p < 0.001; FoMO, fear of missing out; IGD, Internet Gaming Disorder.

### The Mediator of FoMO on Internet Gaming Disorder Under Different Negative Emotions

In order to test the mediating effect of FoMO in depression, anxiety, stress, and Internet gaming disorder, we standardized the data and adjusted for gender, age, grade, game duration, and type of game, using the SPSS data analysis of model 4 in PROCESS. The results showed (Table 3) depression, anxiety, and stress are significantly positively related to FoMO (β = 0.53, *p* < 0.001, β = 0.58, *p* < 0.001, β = 0.59, *p* < 0.001). After introducing FoMO as a mediating variable into the equation, it was found that depression and stress are still significantly positive predictive of Internet gaming disorder (β = 0.16, *p* < 0.05, β = 0.14, *p* < 0.05), but anxiety is not a significantly positive predictor of Internet gaming disorder (β = 0.13, *p* > 0.05). Meanwhile, FoMO as a mediating variable is also significantly positively related to Internet gaming disorder (β = 0.17, *p* < 0.01, β = 0.18, *p* < 0.01, β = 0.17, *p* < 0.01).

**Table 3:** Path Analysis.

	**β**	***SE***	***95% CI***
**Direct path (Direct effects)**			
Depression → IGD	0.15	0.06	[0.04, 0.28]
Anxiety → IGD	0.13	0.06	[−0.001, 0.25]
Stress → IGD	0.14	0.07	[0.01, 0.27]
**Path through FoMO (Indirect effects)**			
Depression → FoMO → IGD	0.09	0.04	[0.02, 0.16]
Anxiety → FoMO → IGD	0.10	0.04	[0.03, 0.18]
Stress → FoMO → IGD	0.10	0.04	[0.02, 0.19]

*FoMO, fear of missing out; IGD, Internet Gaming Disorder*.

**Figure 2:**
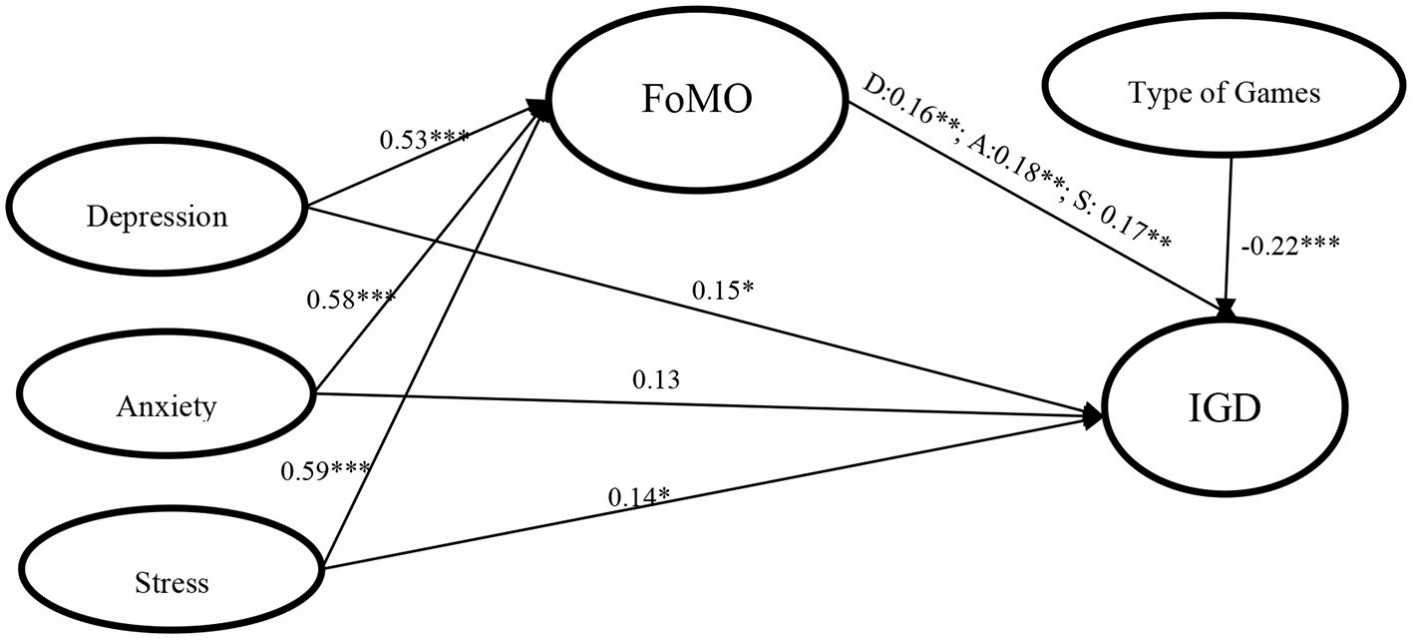
The mediating role of FoMO in the various dimensions of negative emotion and internet gaming disorder. *p < 0.05, **p < 0.01, ***p < 0.001; FoMO, fear of missing out; IGD, Internet Gaming Disorder.

The mediating effect test found that depression, anxiety, and stress are significantly related to Internet gaming disorder through the partial mediating factor of FoMO (*ab* = 0.09, *Boot SE* = 0.04, *95% CI* is [0.02, 0.16], *ab* = 0.10, *Boot SE* = 0.04, *95% CI* is [0.03, 0.18], *ab* = 0.10, *Boot SE* = 0.04, *95% CI* is [0.02, 0.19]), the relative effect of depression, anxiety, and stress are, respectively, 35.45%, 44.95%, and 41.90%. And the mediator relationship of all variables is shown in Figure 2.

## Discussion

This study investigated the relationship of depression, anxiety, stress, and Internet gaming disorder, and explored the role of FoMO in the relationship between depression, anxiety, stress, and Internet gaming disorder. The current study focuses on Chinese teenagers to explore whether there is a difference between the settings of China and the West, and to explore the validity of FoMO scales in cultural adaptability.

This study found that depression, anxiety, and stress are significantly positively correlated to Internet gaming disorder, and FoMO is significantly relevant to anxiety, depression, stress, and Internet gaming disorder, supporting H1 and H2, which is consistent with previous research. Previous studies found that individuals with higher levels of depression, anxiety, and stress are more likely to develop an Internet games disorder (16, 19, 30). A possible reason is that Internet games are a way for individuals to escape from reality. And they can receive sequential rewarding experience during game playing, and increase their craving for Internet games. Therefore, individuals with higher levels of depression, anxiety, and stress are more willing to spend time playing Internet games. Previous studies also found that FoMO has a positive relationship with anxiety and depression severity (18) and Internet gaming disorder (22, 23). One interesting result is that the relationship between FoMO and IGD in this study (*r* = 0.27) is higher than in previous studies (*r* = 0.12) (24), which may be induced by different FoMO scales used in two studies. Although the FoMO scale in this study has a good validity and reliability, and most previous studies on FoMO were conducted in a Western culture setting, FoMO may have different external performances in different contexts, so the results are different.

This study also found that when FoMO acts as a mediating factor, there is still a significant relationship among depression, stress, and Internet gaming disorder, but there is no significant relationship between anxiety and Internet gaming disorder. It means that FoMO acts as a part mediator among depression, stress, and Internet gaming disorder, and acts as a full mediator between anxiety and Internet gaming disorder, supporting H3. Specifically, anxiety, stress, and depression symptoms will increase the level of FoMO (18), and higher FoMO is more likely to result in impulsive behaviors and spending more time playing Internet games (24), Meanwhile, higher FoMO in anxiety symptoms leads to more risk to develop to Internet gaming disorder. The current study found that different types of games have different predictive effects on Internet gaming disorder, as the results showed that the scores of Internet gaming disorder of students who played Honor of Kings or Player Unknown's Battlegrounds are significantly higher than students who played single-player games. A possible reason is that FoMO is a kind of anxiety symptom caused by fear of missing out on the rewarding experience of others (20), while Honor of Kings or Player Unknown's Battlegrounds may provide more opportunities to interact with others and provide more opportunities for continuous rewards. Therefore, anxious individuals with higher FoMO will be more at risk for Internet gaming disorder. According to the I-PACE model, FoMO might create an attention bias in that people are worried about missing reward experiences or other information (26, 27), Internet games might meet the needs of individuals with fear of missing rewards or information and obtain the corresponding psychological satisfaction, so that they develop into Internet gaming disorder.

More interestingly, the results of this study indicated that anxiety and stress have a greater predictive effect on Internet gaming disorder through the mediating relationship of FoMO (44.95%, 41.90%) than depression (33.45%). A possible reason is that depressed individuals pay more attention to themselves and are unwilling to have too much contact with the real world. Though Internet games may make depressed individuals satisfied through socializing or rewarding experiences, other Internet activities (e.g., social networking or online shopping) may also have the opportunity to allow depressed individuals to escape from real world interaction. According to the I-PACE model, the external stimulus provides individuals with clues to rewarding experiences, which can promote cravings for information related to Internet activities, thereby forming Internet addiction (26, 27). As the results of this study found, individuals with different emotional states may develop different cognition or affection bias to Internet activities, and then develop into different Internet addiction. This finding provides the basis for future research on the impact of FoMO on different types of Internet addiction, and provides evidence for the I-PACE model to explain the mechanism of first choice of behaviors to develop specific addictive behaviors.

## Limitations

It cannot be denied that this study has some limitations. Firstly, this study used Chinese students only; it may cause a lower external validity. Future researchers should consider more participants from different culture settings. Secondly, the cross-sectional method cannot reveal the causal relationship among depression, anxiety, stress, FoMO, and Internet gaming disorder. Future researchers may explore it through other methods. Thirdly, the study did not consider the mechanism changes in the brain. Future research can explore the different mechanisms of the brain of FoMO individuals, to validate the interpretation of the I-PACE model in specific types of Internet addiction.

## Conclusion

In sum, this study found that the FoMO scale has a good validity in the context of China, and depression and stress are significantly related to Internet gaming disorder through the partial mediating factor of FoMO. Anxiety is not significantly related to Internet gaming disorder through the fully mediating factor of FoMO. Specifically, under the mediation of fear of missing out, teenagers with anxiety are more likely to develop Internet gaming disorder, while teenagers with depression or stress might be prone to other types of Internet use disorders.

## Data Availability Statement

The original contributions presented in the study are included in the article/Supplementary Material, further inquiries can be directed to the corresponding author/s.

## Ethics Statement

The studies involving human participants were reviewed and approved by Ethics Committee of Tianjin Normal University. Written informed consent to participate in this study was provided by the participants' legal guardian/next of kin.

## Author Contributions

YW and LZ were responsible for the analysis and interpretation of data, drafted the first version of the manuscript, and critically revised the manuscript. BL collected the data for this study. YW, LZ, and PZ provided critical revisions of the manuscript for important intellectual content and approved the final manuscript. All authors approved the final manuscript as submitted and agreed to be accountable for all aspects of the work.

## Funding

This work was supported by the Postgraduate Research and Innovation Project of Tianjin (2021YJSB320).

## Conflict of Interest

The authors declare that the research was conducted in the absence of any commercial or financial relationships that could be construed as a potential conflict of interest.

## Publisher's Note

All claims expressed in this article are solely those of the authors and do not necessarily represent those of their affiliated organizations, or those of the publisher, the editors and the reviewers. Any product that may be evaluated in this article, or claim that may be made by its manufacturer, is not guaranteed or endorsed by the publisher.
